# A Comprehensive Review on Navigating the Neurological Landscape of COVID-19: Insights Into Etiopathogenesis and Clinical Management

**DOI:** 10.7759/cureus.60079

**Published:** 2024-05-11

**Authors:** Roma Chavhan, Anil Wanjari, Sunil Kumar, Sourya Acharya, Nishant Rathod, Harshitha Reddy, Rinkle Gemnani

**Affiliations:** 1 Medicine, Jawaharlal Nehru Medical College, Datta Meghe Institiute of Higher Education and Research, Wardha, IND

**Keywords:** rehabilitation, prognostic implications, clinical management, etiopathogenesis, neurological manifestations, covid-19

## Abstract

The coronavirus disease 2019 (COVID-19) caused by severe acute respiratory syndrome coronavirus 2 (SARS-CoV-2) has emerged as a global health crisis with significant neurological implications. While initially characterized by respiratory symptoms, COVID-19 has been increasingly recognized for its diverse neurological manifestations, including encephalopathy, stroke, peripheral neuropathies, and neuropsychiatric disorders. Understanding the neurological landscape of COVID-19 is essential for elucidating its pathophysiology, optimizing clinical management, and improving patient outcomes. This comprehensive review provides insights into the etiopathogenesis, clinical manifestations, diagnostic approaches, management strategies, and prognostic implications of neurological involvement in COVID-19. Mechanistic insights highlight the multifactorial nature of neurological complications involving direct viral invasion, immune-mediated mechanisms, and thrombotic events. Diagnostic challenges underscore the importance of a multidisciplinary approach to patient care, while management strategies emphasize early recognition and appropriate intervention. Long-term neurological sequelae and prognostic factors are also examined, emphasizing the need for comprehensive follow-up and rehabilitation services. Finally, recommendations for future research prioritize efforts to elucidate underlying mechanisms, identify biomarkers, and evaluate rehabilitative interventions. By addressing these challenges, we can better understand and mitigate the neurological consequences of the ongoing COVID-19 pandemic.

## Introduction and background

The coronavirus disease 2019 (COVID-19), caused by the severe acute respiratory syndrome coronavirus 2 (SARS-CoV-2), has evolved into a global health crisis since its emergence in late 2019 [1]. Initially recognized for primarily respiratory manifestations, COVID-19 has increasingly revealed its multi-systemic nature, including significant neurological involvement. While respiratory symptoms remain the hallmark of the disease, neurological manifestations have garnered attention due to their prevalence, diversity, and potential impact on patient outcomes [2].

The recognition of neurological manifestations of COVID-19 is crucial for several reasons. First, it expands our understanding of the disease's pathophysiology, shedding light on viral neuroinvasion and immune-mediated injury mechanisms [3]. Second, neurological complications can significantly contribute to morbidity and mortality, emphasizing the importance of early recognition and appropriate management. Third, neurological involvement in COVID-19 poses unique diagnostic and therapeutic challenges, necessitating a comprehensive understanding of its diverse presentations and clinical course [4].

This comprehensive review aims to provide insights into the neurological landscape of COVID-19, encompassing its etiopathogenesis, clinical manifestations, diagnostic approaches, management strategies, and prognostic implications. By synthesizing existing literature and emerging evidence, the review seeks to elucidate the complexities of neurological involvement in COVID-19 and offer guidance for clinicians and researchers alike.

## Review

Neurological manifestations of COVID-19

Overview of Neurological Symptoms

**Figure 1:**
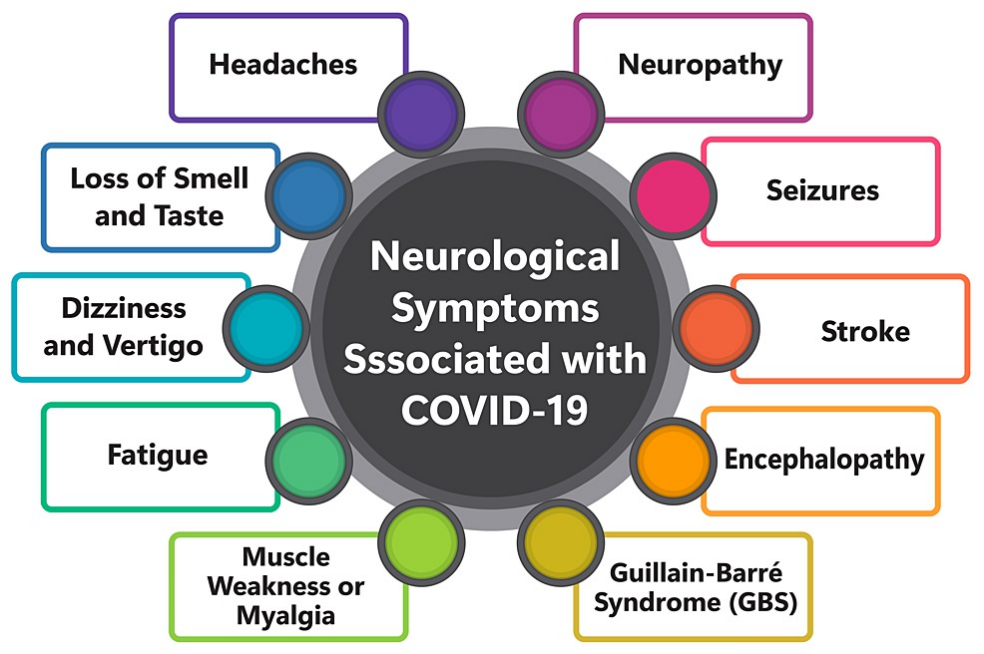
Shows neurological symptoms associated with COVID-19 The image is created by the corresponding author.

The neurological symptoms associated with COVID-19 can be classified into three main categories: central nervous system (CNS) manifestations, peripheral nervous system (PNS) manifestations, and skeletal muscle manifestations. Among the most commonly reported neurological symptoms of COVID-19 are myalgia, headache, altered sensorium, hyposmia, and hypogeusia [5-7]. CNS manifestations encompass conditions such as headache, encephalitis, encephalopathy, and stroke [5,7]. Headache, in particular, is prevalent among patients, with approximately 19.88% experiencing this symptom on average [5]. Some COVID-19 patients with neurological manifestations initially present only with fever and headache, followed by cough, throat pain, lymphopenia, and ground-glass appearance on their chest computed tomography (CT) scans [5]. PNS manifestations involve taste and smell dysfunction [5,7], while skeletal muscle manifestations primarily manifest as myalgia [5,7]. In addition to these symptoms, COVID-19 can lead to cognitive deficits, tremors, and difficulty maintaining balance [6]. A subgroup of individuals may exhibit a distinct set of symptoms called post-acute sequelae of COVID-19 infection with tremors, ataxia, and cognitive deficit (PASC-TAC) [6]. These neurological symptoms often persist in most COVID-19 long-haulers, significantly affecting their quality of life and cognitive functioning [6]. Consequently, understanding and effectively managing the neurological aspects of COVID-19 is imperative for enhancing patient outcomes and minimizing morbidity and mortality. Figure 1 shows neurological symptoms associated with COVID-19.

Types of Neurological Involvement

Central nervous system (CNS) manifestations: The neurological effects of COVID-19 extend to both the central and peripheral nervous systems, encompassing symptoms ranging from headaches and dizziness to ischemic stroke and cerebral hemorrhage [8]. Commonly reported CNS manifestations include myalgia, headache, altered sensorium, hyposmia, and hypogeusia [2]. It's noteworthy that neurological symptoms may precede respiratory manifestations in some instances, underscoring the importance of early recognition and management of neurological complications in COVID-19 patients [5]. The pathophysiology of these neurological manifestations is multifaceted, potentially involving direct viral injury, inflammation, or coagulopathy [8]. Evidence suggests that the SARS-CoV-2 virus can interact with angiotensin-converting enzyme 2 (ACE2) receptors in neurons, potentially leading to viral replication and subsequent neuronal damage [8]. Additionally, inflammatory demyelinating mechanisms, such as acute disseminated encephalomyelitis (ADEM), have been observed in COVID-19 patients [8]. Ongoing research explores the relationship between COVID-19 and neurodegenerative diseases, with some studies indicating a possible association between the virus and the development of such disorders [9].

Peripheral nervous system (PNS) manifestations: Peripheral nervous system manifestations in COVID-19 patients encompass a spectrum of conditions, including nerve pain, skeletal muscle injury, Guillain-Barré syndrome, cranial polyneuritis, neuromuscular junction disorders, neuro-ophthalmological disorders, neurosensory hearing loss, and dysautonomia. These manifestations may arise from dysregulation of the immune system triggered by COVID-19. Guillain-Barré syndrome is the most commonly reported PNS manifestation, accounting for 84.2% of PNS events in COVID-19 patients. Among cranial nerves, the facial, vestibulocochlear, and olfactory nerves are most frequently affected in COVID-19 patients, with involvement rates of 27.3%, 25.4%, and 16.1%, respectively [10]. PNS involvement in COVID-19 can significantly contribute to hospitalization rates and post-COVID-19 sequelae, thereby increasing healthcare systems' burden [11]. Notably, patients with PNS involvement tend to experience more severe COVID-19 disease, with 37.3% of them classified as having severe disease [11]. The most common neurological outcome in patients with CNS, PNS, and combined PNS and CNS involvement is mild to moderate sequelae, with no significant differences observed in mortality rates, disease severity, duration from disease onset to neurological symptoms, lack of improvement, or complete recovery among the three categories [10].

Neuropsychiatric manifestations: Neuropsychiatric symptoms are prevalent among COVID-19 patients and can significantly impact prognosis and mortality risk [12,13]. These symptoms encompass a wide range, including anxiety, mood disorders, headaches, sleep disturbances, encephalopathy, stroke, and other neurological conditions [12,13]. The presence of neuropsychiatric symptoms in COVID-19 patients may be attributed to neurotropic mechanisms, with emerging evidence suggesting that SARS-CoV-2 exhibits neurotropic properties that can lead to neurological damage [13]. A recent study revealed a notably higher incidence of psychiatric disorders among patients recovering from COVID-19, with a 5.8% probability of newly diagnosed psychiatric illness within 90 days post-diagnosis [13]. Anxiety disorder was the most commonly diagnosed psychiatric condition, followed by mood disorders [13]. Furthermore, pre-existing psychiatric conditions were independently associated with an increased risk of COVID-19 diagnosis [13]. The precise mechanism by which SARS-CoV-2 induces neuropsychiatric manifestations remains incomplete, although hypotheses point to neuroinflammation, neuroinvasion, and disruption of neurotransmitter systems [13]. The virus can directly infect the nervous system, causing neurological damage, or indirectly impact it through systemic inflammation and immune responses [13]. Recognizing and managing these neuropsychiatric complications is crucial for improving patient outcomes and reducing mortality rates [14]. Healthcare providers should actively screen for neuropsychiatric symptoms in COVID-19 patients and offer appropriate interventions such as psychotherapy, pharmacotherapy, and rehabilitation [2]. Furthermore, further research is warranted to elucidate the underlying mechanisms and risk factors associated with neuropsychiatric manifestations in COVID-19 patients, which can inform preventive and therapeutic strategies [14].

Epidemiology of Neurological Complications

The epidemiology of neurological complications in COVID-19 patients has been the subject of extensive study. Reported prevalence rates of neurological symptoms among COVID-19 patients range widely from 9.9% to 65% [15]. In a study involving 219 COVID-19 patients from a single center, acute cerebrovascular events (CVEs) were observed in 4.6% of cases, with nine ischemic events and one hemorrhagic stroke recorded [16]. Another investigation of 49 COVID-19 patients noted a mortality rate of 15%, with fever being the most commonly reported symptom [15]. A retrospective analysis of 561 COVID-19 patients from a Mexican center highlighted headache, neurological impairment, ageusia, and anosmia as the most prevalent neurological manifestations, accounting for 80% of cases [17]. Notably, neurological impairment, either upon admission or before hospitalization, was identified as a significant risk factor for mortality among these patients. In a prospective study involving 355 individuals, hyposmia and cough were associated with a 5.46 odds ratio for COVID-19 infection [18]. Of note, subjective hyposmia and hypogeusia were reported in 138 cases (64.1%) and 114 cases (53%), respectively, with 85.4% of patients experiencing recovery of olfactory function within the first 14 days of symptom onset [18].

Etiopathogenesis of neurological involvement

Mechanisms Underlying Neurological Manifestations

Direct viral invasion: Understanding the etiopathogenesis of neurological involvement in COVID-19 involves grappling with the complexities of direct viral invasion and maladaptive inflammatory responses. Direct viral invasion stands out as one mechanism underlying neurological manifestations in COVID-19. However, definitive evidence supporting central nervous system (CNS) invasion by the COVID-19 virus remains elusive, with no large-scale studies conducted to substantiate or refute this claim [19]. The virus can potentially breach the CNS through various routes, including the hematogenous, neuronal retrograde, and olfactory transmucosal routes [20]. In the hematogenous route, the virus gains access to the CNS via systemic circulation, potentially crossing the blood-brain barrier (BBB) [20]. Conversely, the neuronal retrograde route involves viral migration from the peripheral nervous system to the CNS through nerve fibers [20]. Furthermore, the olfactory transmucosal route entails the virus infiltrating the CNS via the olfactory nerve within the nasal cavity [20]. The presence of acute infarcts in the brainstem and detection of intact CoV particles at the ultrastructural level within the CNS endothelium suggests possible SARS-CoV-2 neuroinvasion occurring at the neural-mucosal interface through transmucosal entry via the olfactory tract, offering insight into documented neurological symptoms such as alterations in smell and taste perception [4]. However, the interpretation of morphological and molecular analyses may be constrained by cellular and tissue autolysis, particularly under emergency-like conditions encountered during a pandemic [20].

Indirect effects of systemic inflammation: The impact of systemic inflammation on the neurological landscape of COVID-19 is profound and multifaceted. Systemic inflammation, arising from a perpetually vigilant immune system, triggers the release of proinflammatory cytokines and chemokines, serving as immune mediators that incite inflammatory responses across the body [21]. This systemic inflammation can disrupt physical and mental function, particularly when it perturbs the CNS equilibrium [21]. In COVID-19, systemic inflammation can precipitate neurological manifestations such as encephalopathy, encephalitis, myelitis, Guillain-Barré syndrome, and various peripheral nervous system disorders [22]. Possible mechanisms of neurological injury encompass direct viral invasion, inflammation, and coagulopathy [22]. Recognizing the impact of COVID-19 on chronic neurological conditions is imperative, prompting proactive measures from treating clinicians to mitigate pandemic-related disruptions and assuage patient and caregiver concerns [21]. The interaction between the immune system and CNS, comprising the brain and spinal cord, is mediated by the blood-brain barrier, which maintains separation between the systems and safeguards the brain's balanced environment [21]. However, systemic inflammation can induce subtle alterations in the blood-brain barrier, permitting entry of proinflammatory cytokines and chemokines into the brain. Consequently, the brain's inflammatory response may be triggered, precipitating cognitive and behavioral symptoms such as memory lapses, confusion, and sickness behavior characterized by depression, decreased physical activity, fatigue, lack of motivation, and appetite disturbances [21].

Hypoxic injury and thrombotic complications: Hypoxic injury and thrombotic complications pose significant risks to COVID-19 patients. The virus can precipitate thrombotic events, including venous thromboembolism (VTE), stroke, and acute myocardial infarction [23]. Underlying pathophysiological mechanisms involve the activation of coagulation pathways, endothelial dysfunction, and inflammation [23]. Hypoxia, a prevalent symptom in COVID-19 patients, can exacerbate these complications by promoting thrombosis and enhancing inflammatory cytokine production [24]. Diagnosing thrombotic complications in COVID-19 patients can be challenging due to symptom overlap between pulmonary embolism and severe COVID-19 illness [23]. Nonetheless, early recognition and vigilant monitoring of coagulation abnormalities are imperative for identifying these complications, guiding antithrombotic prevention or treatment, and enhancing patient outcomes [23]. Most guidelines and consensus documents issued by professional societies recommend thromboprophylaxis for hospitalized COVID-19 patients, particularly those at high risk of VTE post-discharge and without bleeding risk factors [23]. Hypoxia can also precipitate thrombotic sequelae and organ dysfunction in long-term COVID-19, exacerbating vascular inflammation and coagulation abnormalities [24]. Early prophylactic anticoagulation may mitigate procoagulant substance release or removal, safeguarding vascular endothelium, reducing thrombotic sequelae, and enhancing the quality of life for long-COVID patients [24].

Immune-mediated mechanisms: Immune-mediated mechanisms play a pivotal role in the neurological manifestations of COVID-19. These mechanisms encompass heightened cytokine levels, blood-brain barrier compromise, immune cell infiltration, vascular inflammation, and vessel occlusion [25]. The immune response to SARS-CoV-2 infection may be dysregulated, leading to unchecked immunity, culminating in pulmonary tissue damage, functional impairment, and diminished lung capacity [26]. Conversely, immune insufficiency or misdirection may foster viral replication and tissue injury [26]. Beyond macrophages and monocytes, the innate immune response to SARS-CoV-2 often involves abnormal activation and recruitment of neutrophils, with severe COVID-19 stages characterized by a notable increase in myeloid-derived suppressor-like cells (MDSC-like) that hinder viral clearance and suppress T cell function [27]. Neutrophils, known for releasing neutrophil extracellular traps (NET), contribute to pathophysiology by trapping inflammatory cells and impeding tissue repair cell recruitment [27-28]. Strategies aimed at modulating NET formation or degrading NET using agents like DNase hold promise as potential therapies for severe COVID-19 cases [27-30].

Neurological complications associated with COVID-19

Encephalopathy and Delirium

Encephalopathy and delirium emerge as notable neurological complications associated with COVID-19. Encephalopathy is characterized by varying degrees of altered consciousness, from mild confusion and delirium to profound coma, while delirium primarily manifests as acute disturbances in attention, awareness, and cognition [31]. An analysis of ICU patients with severe COVID-19 revealed that 84.3% developed delirium, with 63.6% exhibiting corticospinal tract signs suggestive of encephalopathy [32]. This study further noted that delirium and neurological symptoms in COVID-19 patients correlated with prolonged mechanical ventilation compared to those without such symptoms [32]. The etiology of delirium and encephalopathy in COVID-19 remains incompletely understood. Immunological dysregulation and redox imbalance are proposed contributors to neurological symptom development in COVID-19 [32]. Leveraging bioinformatics to analyze extensive patient datasets and develop novel disease models holds promise in identifying new risk factors and treatment targets [32]. Additionally, postmortem neuropathological studies and baseline neurological assessments are deemed indispensable for comprehending the neurological ramifications of COVID-19 [32]. Further investigation is warranted to discern whether delirium in COVID-19 signifies a primary encephalopathy heralding viral invasion of the CNS or a secondary complication stemming from systemic inflammation [33]. The frequent occurrence and reproducibility of neurological signs in COVID-19 patients suggest that the virus may underlie at least some cases of delirium and encephalopathy [32]. However, it is imperative to rule out other potential causes of delirium/encephalopathy, such as iatrogenic, alcoholic, or metabolic factors, before attributing symptoms solely to COVID-19 [32].

Stroke and Cerebrovascular Events

Stroke and cerebrovascular events have emerged as significant complications associated with COVID-19 infection, with mounting evidence indicating a notable risk of thrombotic events, including stroke, in afflicted patients [34,35]. The incidence of cerebrovascular events among COVID-19 patients is relatively elevated, with an odds ratio of 7.6 compared to individuals with influenza [36]. These events manifest in diverse forms, encompassing both ischemic and hemorrhagic presentations, each with distinct pathophysiological underpinnings [36]. While the etiology of cerebrovascular events in COVID-19 patients may not always be straightforward, the disease is frequently associated with a proinflammatory and procoagulant state that may predispose individuals to such complications [36]. In certain instances, COVID-19 infection has been explicitly implicated as a potential etiological cofactor, particularly in conjunction with known risk factors like estrogen-progestin therapy [36]. Reported cases of ischemic stroke in COVID-19 patients commonly exhibit pre-existing risk factors such as hypertension, atrial fibrillation, vascular disease, diabetes, and smoking [36]. Remarkably, the age distribution of ischemic stroke patients within COVID-19 cohorts closely mirrors that of individuals at higher risk for stroke, with an average age of 62.9 ± 17.2 years and a median age of 67.5 years [36]. A temporal association between COVID-19 and cerebrovascular events is evident in all reported case accounts, with a hypothesized etiopathogenetic link proposed between COVID-19-related coagulopathy and stroke in select instances [36]. Managing cerebrovascular events in COVID-19 patients demands a multidisciplinary approach, incorporating antithrombotic therapy, mechanical thrombectomy, and intravenous thrombolysis [37]. Nonetheless, the optimal duration and type of antithrombotic treatment for patients with stroke presumed to be related to COVID-19 remain uncertain, necessitating further investigation to elucidate the most effective therapeutic strategies [37].

Encephalitis and Meningitis

Encephalitis and meningitis affect the central nervous system, characterized by inflammation of the brain and the membranes surrounding the brain and spinal cord, respectively [38,39]. While both can stem from viral or bacterial infections, their symptoms may overlap, including fever, headache, vomiting, and a stiff neck and back [38,39]. However, encephalitis may present symptoms such as confusion, impaired judgment, drowsiness, muscle weakness, unsteady gait, and irritability, while meningitis may manifest as changes in behavior, sleepiness, and difficulty awakening [39]. Meningoencephalitis, a rare and life-threatening condition, entails concurrent inflammation of both the brain and meninges, necessitating prompt treatment [40]. Treatment for encephalitis and meningitis hinges on the underlying cause and severity. Antiviral medications, such as those prescribed for herpes encephalitis or severe viral infections and antibiotics for bacterial infections, are often utilized [39]. Anticonvulsants may prevent or manage seizures, while corticosteroids can mitigate brain swelling and inflammation [39]. Sedatives may address irritability or restlessness; over-the-counter medications may alleviate fever and headache symptoms [39]. Vaccination is a preventive measure against specific types of meningitis or encephalitis, with vaccines like pneumococcal, meningococcal, *Haemophilus influenzae* b, and Japanese encephalitis vaccines available [38]. Severe cases of encephalitis or meningitis may precipitate complications such as loss of muscle control, sensory loss, partial paralysis, hearing or speech impairments, blindness, permanent neurological damage, alterations in behavior or personality, memory loss, cognitive impairments, seizures, and even death [38]. Prognosis hinges on disease severity and prompt diagnosis and treatment initiation, as severe cases may rapidly progress, leading to irreversible neurological deficits or fatalities [38].

Guillain-Barré Syndrome and Other Peripheral Neuropathies

Guillain-Barré syndrome (GBS) is an acute, swiftly progressing, and typically self-limiting inflammatory polyneuropathy characterized by muscular weakness and mild sensory loss, primarily affecting the distal extremities [41]. It is believed to have an autoimmune etiology, with diagnosis primarily relying on clinical presentation [41]. Treatment modalities encompass intravenous immune globulin (IVIG), plasma exchange, and, in severe cases, mechanical ventilation [2]. GBS is the most prevalent acquired inflammatory neuropathy, exhibiting various subtypes, some predominantly affecting the myelin sheath, while others primarily target the axon [41]. The prognosis for GBS is generally favorable, with less than 4% of patients succumbing to the disease [41]. Nonetheless, a considerable proportion of adults, and even more children, may experience residual weakness persisting up to three years post-onset, with approximately 5% transitioning to chronic inflammatory demyelinating polyneuropathy (CIDP) [41]. Patients with residual deficits may necessitate rehabilitation, orthopedic interventions, or surgical procedures [41]. Accurate differentiation of GBS from inherited neuropathies and other acquired peripheral neuropathies entails recognizing the atypical presentations of GBS and its variant forms, alongside considering historical and physical indicators suggestive of inherited neuropathies [42]. GBS typically manifests with the sudden onset of ascending flaccid paralysis, absence of reflexes, and sensory disturbances attributable to demyelination of peripheral nerve fibers [42]. Although the diagnosis of GBS primarily relies on clinical evaluation, electrodiagnostic testing, and cerebrospinal fluid (CSF) analysis may offer supplementary confirmation [42]. Treatment approaches encompass IVIG, plasma exchange, and, in severe cases, mechanical ventilation. Emphasizing intensive supportive care is pivotal for fostering recovery, with a stepwise approach favoring initial IVIG administration, followed by plasma exchange if deemed ineffective [42].

Neuropsychiatric Disorders

Neuropsychiatric disorders have emerged as notable complications associated with COVID-19 infection, as indicated by several large-scale electronic health records studies reporting elevated diagnostic rates for these conditions [43]. The risks of developing neuropsychiatric disorders after COVID-19 infection appear to be linked to the severity of the illness and can endure for an extended duration [43]. Among the most prevalent neuropsychiatric disorders following COVID-19 infection are mood disorders, anxiety disorders, psychotic disorders, and cognitive impairment, colloquially referred to as "brain fog" [43]. Compared to other respiratory infections or health events, the risks of these disorders are notably higher post-COVID-19 infection, with particularly elevated risks observed for psychotic disorder and brain fog, surpassing those for mood and anxiety disorders [43]. While the precise mechanisms underlying these neuropsychiatric disorders after COVID-19 infection remain incompletely understood, potential contributors may include microvascular disease, metabolic dysregulation, general inflammation, and drug toxicity or side effects [44]. Advanced magnetic resonance imaging (MRI) studies have revealed abnormalities consistent with widespread brain damage, including in crucial brainstem arousal nuclei, among patients exhibiting persistent unresponsiveness following COVID-19 infection [44]. In light of these findings, maintaining neuroprotective measures in COVID-19 patients is imperative, especially given that high exposure to sedatives, particularly benzodiazepines, has been independently associated with elevated rates of delirium [44].

Long-Term Neurological Sequelae

The long-term neurological ramifications of COVID-19 pose significant concerns for many patients, with a diverse array of symptoms reported in various studies. A meta-analysis revealed that individuals with severe COVID-19 manifestations faced elevated odds of experiencing persistent symptoms such as headache, fatigue, myalgia, anosmia, and dysgeusia long after the acute phase of the infection [45]. Moreover, another study indicated that long-lasting neurological complications could impact up to 45% of both hospitalized and non-hospitalized COVID-19 survivors, with fatigue and cognitive complaints emerging as the most prevalent issues [46]. Furthermore, there have been associations between COVID-19 and neurodegenerative complications like parkinsonism and dementia, with heightened risks observed within the six months following infection [3]. A study highlighted a notable decline in cognitive function among individuals infected with SARS-CoV-2, as evidenced by a four-point reduction in the Montreal Cognitive Assessment (MoCA) from pre-pandemic to post-pandemic evaluations [46]. Furthermore, neurological symptoms after COVID-19 have been linked with alterations in brain microstructure observed on imaging examinations. This correlation is supported by evidence from various studies, encompassing in vivo, in vitro, and animal research, suggesting the potential neuroinvasive nature of the novel COVID-19 virus [46]. Additionally, specific prodromal indicators, such as impaired olfaction and rapid eye movement (REM) sleep behavior disorder, have been identified as potential predictors of Parkinson's disease and dementia [46]. These findings underscore the need for continued vigilance and comprehensive monitoring of neurological sequelae in individuals recovering from COVID-19 infection.

Diagnostic approaches

Challenges in Diagnosing Neurological Complications

**Figure 2:**
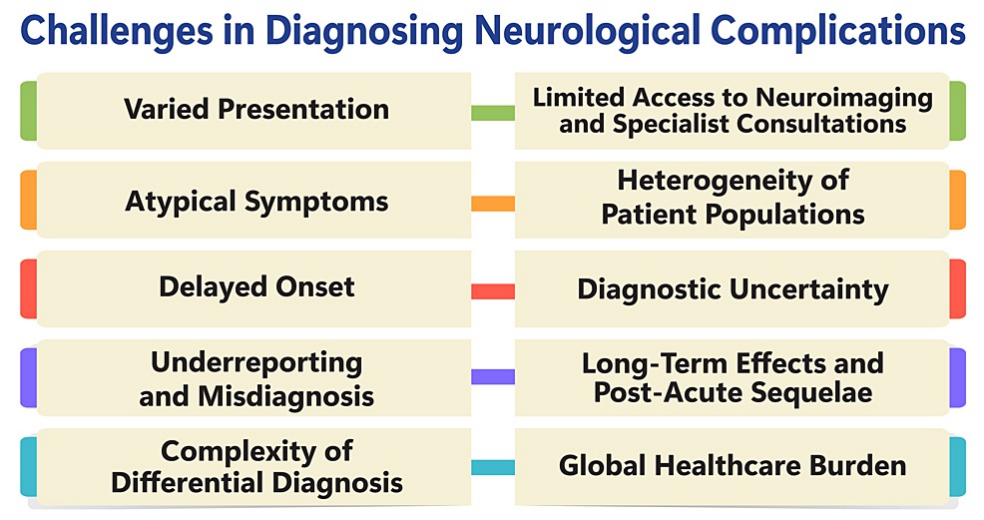
Shows challenges in diagnosing neurological complications The image is created by the corresponding author.

Diagnosing neurological complications presents formidable challenges due to the intricate nature of the nervous system and the broad spectrum of disorders that can affect it. These disorders pose significant burdens on healthcare systems globally, impacting individuals' quality of life through physical, emotional, and cognitive impairments [47]. In many real-world scenarios, limited resources often result in insufficient care for patients with neurological disorders, exacerbating challenges in diagnosis and treatment and potentially leading to misdiagnosis and delayed care [47]. The complexity of neurological conditions, ranging from Alzheimer's disease to Parkinson's disease, epilepsy, stroke, and headaches, renders accurate diagnosis and effective treatment particularly challenging. These disorders manifest through diverse symptoms, necessitating specialized care, diagnostic tools, and intricate treatment options, further complicating their management [47,48]. Misdiagnosis and delayed care can exacerbate symptoms and place additional strain on patients and caregivers, underscoring the urgent need for improved access to care, research advancements, and public awareness surrounding neurological disorders [47]. Furthermore, the substantial volume and complexity of medical data generated by diagnostic technologies like magnetic resonance imaging and electroencephalograms present a significant challenge for experts to analyze manually. In response, the demand for computer-aided diagnosis (CAD) systems has emerged, aiming to detect neurological abnormalities in vast medical datasets automatically. These systems offer potential solutions to enhance diagnostic consistency, treatment efficacy, and patient outcomes [49]. Figure 2 shows challenges in diagnosing neurological complications.

Neuroimaging Modalities

Neuroimaging modalities play a crucial role in diagnosing and managing neurological manifestations in COVID-19 patients, with brain MRI being the most commonly utilized modality in clinical practice. According to a systematic review, brain MRI was employed in seven of ten studies [50]. The recommended MRI protocol typically includes a combination of T2-weighted, FLAIR (preferably 3D), and diffusion-weighted images, along with hemorrhage-sensitive sequences (preferably SWI) and pre-and post-contrast T1 weighted-images for the initial investigation [51]. To optimize the detection of leptomeningeal contrast, acquiring 3D FLAIR after gadolinium administration is recommended [51]. In cases where COVID-19 patients present with clinical stroke symptoms, brain CT may be the preferred initial imaging modality to rule out hemorrhage. Additionally, CT, when utilized alongside MRI, can aid in detecting other neurological manifestations such as encephalitis, meningitis, and myelitis [51]. Furthermore, neuroimaging modalities beyond MRI hold unique potential in shedding light on the metabolic and inflammatory pathology of COVID-19. Positron emission tomography (PET) can effectively detect neuroinflammation and neurodegeneration in COVID-19 patients, while single-photon emission computed tomography (SPECT) offers valuable insights into cerebral perfusion and metabolism [52]. Leveraging these advanced imaging techniques can provide clinicians with a comprehensive understanding of neurological complications in COVID-19 and aid in guiding appropriate management strategies.

Laboratory Investigations

Laboratory investigations play a pivotal role in diagnosing COVID-19, with viral detection through nucleic acid amplification tests (NAAT) of respiratory specimens being considered the gold standard [53,54]. The World Health Organization (WHO) recommends diagnosing COVID-19 based on the detection of unique viral RNA sequences using NAAT, particularly real-time reverse transcription-polymerase chain reaction (RT-PCR) [55]. Similarly, the Indian Council of Medical Research (ICMR) advocates RT-PCR as the primary method for detecting SARS-CoV-2 in respiratory samples [55]. In addition to NAAT, serological assays and CT scans are also utilized for COVID-19 diagnosis [1][2]. The ICMR has developed and validated the "COVID KAVACH ELISA", an indigenous IgG enzyme-linked immunosorbent assay (ELISA) test for antibody detection in COVID-19 [55]. Furthermore, the ICMR has issued comprehensive guidelines for specimen collection, packaging, and transport, emphasizing the use of personal protective equipment and biosafety level two or three facilities [55]. Additionally, the ICMR has introduced guidelines for RT-PCR-based pooled sampling, particularly for migrants/returnees from abroad/green zones, wherein a pool of samples is tested, and individual samples within the pool are retested if the pool tests positive [55]. Moreover, the ICMR has assessed the performance of commercial kits for real-time PCR for COVID-19 through validation centers identified by the Institute. However, it's essential to recognize that the sensitivity and specificity of these diagnostic tests may vary, and their utilization should be guided by clinical judgment and resource availability [53].

Clinical Assessment Tools

Clinical assessment tools are crucial in guiding healthcare professionals through assessing and managing patients with COVID-19. One such tool is the COVID-19 Clinical Assessment Tool from WCH CovidCare@Home, a PDF resource that facilitates virtual visits with COVID-19 patients [56]. The Practice Tool #1 - Assessment Guide for Clinicians from the BC COVID Therapeutics Committee provides a step-by-step clinical assessment framework for healthcare providers managing patients with mild to moderate COVID-19 [57]. NHS England Digital offers the COVID-19 Clinical Risk Assessment Tool, powered by QCovid®, which assists clinicians in understanding the risk level of individuals for COVID-19-related mortality following infection [58]. Another valuable resource is the Online Tool for the Assessment of the Burden of COVID-19 in Patients, developed to evaluate the long-term impact of COVID-19 on patients [59]. Furthermore, Queensland Health provides the COVID-19 Clinical Screening and Risk Assessment tool, which aids in assessing the clinical and symptom risk of COVID-19 in patients [60]. These tools are specifically designed to support healthcare professionals in accurately assessing and managing COVID-19 patients, as well as identifying individuals at a higher risk of severe illness or mortality due to the virus. They provide structured frameworks and guidelines to enhance the quality of care and optimize patient outcomes during the ongoing pandemic.

Management strategies

General Principles of Management

Managing neurological complications in COVID-19 patients involves a comprehensive approach encompassing pharmacological and non-pharmacological interventions. Key strategies include the administration of COVID-19 vaccines and antiviral medications such as remdesivir and dexamethasone, which have shown efficacy in reducing the frequency of neurological complications, including stroke, seizures, and meningitis [61,62]. A multidisciplinary approach is essential, with collaboration among neurologists, intensivists, and other healthcare professionals. Early detection and treatment of conditions like meningoencephalitis are crucial to prevent potentially fatal complications such as hemorrhagic encephalopathy [62]. Healthcare providers should remain vigilant for known and unknown neurological complications that may arise in COVID-19 patients. Non-pharmacological interventions, including telemedicine and remote monitoring, are increasingly vital in managing neurological complications during the pandemic. Telemedicine enables healthcare teams to provide continuous care and monitoring while minimizing the risk of viral transmission. Despite challenges in implementation, telemedicine has become necessary for ensuring the provision of neurological care amidst the pandemic [63]. Various adaptations, such as telephone consultations, remote monitoring, and prioritization of patients with urgent neurological conditions, have been implemented to maintain the quality of care for patients with neurological complications during the COVID-19 pandemic [64]. These innovative approaches have helped healthcare systems adapt to the challenges posed by the pandemic while ensuring continued access to essential neurological care for patients in need.

Specific Treatment Approaches for Neurological Complications

Pharmacological interventions: The pharmacological strategies for managing COVID-19, as outlined in the provided sources, encompass several pivotal medications that have demonstrated effectiveness in combating the disease. Tocilizumab, plasma exchange, and steroids have exhibited promising outcomes among COVID-19 patients, with completed clinical trials confirming their efficacy in combating the virus [65]. Additionally, hydroxychloroquine, azithromycin, and arbidol have undergone scrutiny for their potential therapeutic benefits against COVID-19, with arbidol exhibiting efficacy in molecular docking studies by impeding the trimerization of the SARS-CoV-2 spike glycoprotein [66,67]. The narrative review on pharmacological treatment for COVID-19 underscores the ongoing global endeavors to develop efficacious medications and vaccines against the virus. Despite some medicines and vaccines receiving emergency use authorization based on preliminary findings, a definitive treatment regimen for COVID-19 remains elusive, underscoring the complexity of managing the disease [68].

Supportive care measures: Supportive care measures for individuals with COVID-19 encompass a spectrum of interventions to alleviate symptoms and prevent complications, particularly in the early stages of the disease [1]. For patients with mild COVID-19, supportive care measures may entail rest, hydration, temperature monitoring, consultation with healthcare providers regarding over-the-counter medications, observance of respiratory etiquette, maintenance of physical distance from family members, hand hygiene, surface disinfection, self-isolation, avoidance of utensil sharing, and consideration of disposable products for meals [69]. For those with severe COVID-19, supportive care measures may involve oxygen therapy, intravenous fluids, fever and cough management medications, and other interventions to uphold organ function. In certain instances, hospitalization and more intensive therapies such as mechanical ventilation or extracorporeal membrane oxygenation (ECMO) may be warranted [70]. Supportive care is equally critical for patients with underlying medical conditions like cancer, heart disease, or lung disease, who face a heightened risk of severe COVID-19. Such patients may necessitate additional interventions, such as blood transfusions or treatments for their underlying conditions [71]. Telemedicine and remote monitoring represent valuable modalities for managing COVID-19 patients, especially those with mild or moderate symptoms not requiring hospitalization, thereby mitigating transmission risk while ensuring appropriate care delivery and alleviating strain on healthcare systems [72].

Rehabilitation and long-term care: Rehabilitation constitutes a pivotal component in managing neurological complications among COVID-19 patients, to enhance respiratory and cardiac function and forestall long-term neurological sequelae [73]. Incorporating rehabilitation into the treatment paradigm for COVID-19 patients is recommended to facilitate recovery and mitigate disability, particularly in post-acute COVID-19 cases [73,74]. The establishment of COVID-19 rehabilitation units, ranging from high-complexity to low-complexity settings, is imperative to address the diverse needs of patients grappling with neurological disorders, post-stroke conditions, traumatic brain injuries, and related issues [73]. Long-term care for COVID-19 patients contending with neurological complications necessitates a multidisciplinary approach involving specialists in physical and rehabilitation medicine to address cognitive function, mental health, and physical impairments arising post-illness [73]. Post-intensive care syndrome (PICS) and post-acute sequelae of COVID-19 (PASC) can exert substantial impacts on function and quality of life, underscoring the importance of sustained rehabilitation and support for affected individuals [73]. Critical care recovery clinics, offering in-person and telehealth services, have emerged as invaluable resources for ICU survivors, furnishing multidisciplinary care to address the medical and rehabilitation needs of patients grappling with long-term neurological complications post-COVID-19 [74,75].

Multidisciplinary Approach to Management

The neurological effects of COVID-19 can have enduring consequences for recovery and morbidity, impacting individuals who were hospitalized as well as those managed outside hospital settings. These symptoms may persist beyond the acute phase of COVID-19, extending beyond three to four weeks from the initial onset of the illness. For a diagnosis of neuro-PASC (post-acute sequelae of SARS-CoV-2 infection), the persistence or emergence of neurological symptoms attributable to the virus should persist beyond this timeframe [76,77]. Diagnosing and managing the neurological sequelae of COVID-19 demands a multidisciplinary approach involving various medical specialists, including internists, neurologists, psychiatrists, rehabilitation experts, and primary care providers. Effective coordination and communication within healthcare systems are essential to ensure comprehensive care for affected individuals [76,77]. In addition to pharmacological interventions, tailored rehabilitation programs and innovative cognitive therapy protocols are integral to neuro-PASC management. Early identification of affected individuals is crucial to initiate appropriate and timely interventions. Awareness about PASC is growing among the general population and healthcare professionals, but further efforts are needed to comprehend and address this evolving challenge [76].

Future directions and research priorities

Understanding the role of repeated media consumption around COVID-19 in amplifying distress and its impact on mental health is crucial. Establishing representative populations, real-time data collection methods, and involving patients and the public in research is essential for this endeavor [78]. Further investigations into the origins of the COVID-19 outbreak are necessary, as India has joined a growing number of countries demanding a comprehensive investigation [79]. Investigating the neurological impact of COVID-19, including how the brain is affected during a COVID-19 infection, is essential for understanding the disease [80]. Researching the potential of nanobodies produced by a llama's immune system for testing and treating COVID-19 in humans is a promising area of investigation [80].

Understanding the body's response to the virus and developing a protective response is crucial for effective treatments and vaccines [80]. Reducing the prevalence of acute respiratory distress syndrome (ARDS) to save lives is an essential area of research for improving patient outcomes [80]. Investigating the structural secrets of SARS-CoV-2 and using findings to design therapies and treatments is a crucial area of research for developing effective treatments [80]. Developing strategies to prevent COVID-19 infections in the future, including developing a vaccine, is crucial for controlling the disease [80]. Improving diagnostics and surveillance for COVID-19 in children is essential for understanding how the virus is transmitted and developing effective treatments [80].

Understanding the epidemiology of COVID-19 in Seattle Children's patients and healthcare workers, as well as population disparities and the psychological impact of the disease, is essential for developing effective interventions [80]. Investigating the psychological impact of the COVID-19 pandemic on children and families and developing interventions to support their well-being is crucial for addressing the mental health consequences of the disease [81]. Understanding the impact of the COVID-19 pandemic on healthcare workers and developing interventions to support their well-being is essential for maintaining a strong healthcare system [81]. Developing scientifically backed resilience coaching programs for patients, families, and staff is crucial for addressing the mental health consequences of the disease [81]. Investigating the rates of acne vulgaris in post-COVID healthcare workers who have used face masks for prolonged periods is an essential area of research for understanding the health consequences of the disease [81].

## Conclusions

In conclusion, our comprehensive review has illuminated the intricate interplay between COVID-19 and neurological manifestations, revealing a spectrum of clinical presentations and underlying mechanisms. From encephalopathy to peripheral neuropathies, the neurological impact of COVID-19 spans a wide range, reflecting the virus's ability to affect both the central and peripheral nervous systems. Mechanistic insights have underscored the complex interplay of direct viral invasion, immune-mediated responses, and thrombotic events in the pathogenesis of neurological complications. These findings carry significant implications for clinical practice, emphasizing the importance of heightened awareness among healthcare professionals and adopting a multidisciplinary approach to patient care. Early recognition and appropriate management of neurological involvement are paramount for optimizing outcomes and reducing long-term morbidity. Future research endeavors should focus on unraveling the underlying mechanisms of neuroinvasion, identifying biomarkers for early diagnosis and prognostication, and evaluating the efficacy of rehabilitative interventions. Collaborative efforts across disciplines and institutions will be essential in advancing our understanding of COVID-19 neurology and addressing the challenges posed by neurological complications in the ongoing pandemic.
